# Response to second line antiretroviral therapy in India

**DOI:** 10.1186/1471-2334-12-S1-P40

**Published:** 2012-05-04

**Authors:** Anup Singh, Jaya Chakravarty, Anoop Gupta, Shyam Sundar, Madhukar Rai, Avinash Singh, Amit Agarwal

**Affiliations:** 1 Department of Medicine, Institute of Medical Sciences, Banaras Hindu University, Varanasi, UP, India

## Background

Second line antiretroviral therapy (ART) was rolled out in India in December 2008. However, there has been no study to assess the response to second line ART in India. This study aims to see the response to the NACO recommended second line therapy over 24 week period.

## Methods

Study was conducted in ART centre, IMS, BHU. Patients who failed on first line ART (NACO criteria) were included in the study after consent. Virological, immunological and clinical response was assessed at 24 weeks. Independent t-test /Mann-Whitney U test was used for making comparison among the independent groups. Chi-square test was used to test the association between categorical variables. Spearman correlation coefficient was used to measure the degree of association between two variables.

## Results

Out of 78 patients, only 39 (50%) had virological suppression (viral load<47 copies/ml) and 20 (25.6%) had partial response (viral load >47-1000), 10 (12.8%) had viral load >1000 copies/ml and 8 (10.3%) patients died before 6 months and 1 patient was lost to follow up. Median increase of CD4 count was 133 cells /ml (IQR -46-498). Among patients who expired 90% had clinical failure at baseline, 50% and 18.75% were in stage IV and III respectively.

## Conclusion

Currently recommended second line regimen appears to be inadequate as large proportion of patients failed to achieve the desired virological suppression. Our finding implies a relook at the present second line regimen recommended by NACO.

